# Experience of gender-based violence among high school-going youth in Estcourt, KwaZulu-Natal

**DOI:** 10.4102/hsag.v30i0.2935

**Published:** 2025-05-30

**Authors:** Thobile B. Mchunu, Nelisiwe Khuzwayo

**Affiliations:** 1 Discipline of Public Health Medicine, School of Nursing and Public Health, University of KwaZulu-Natal, Durban, South Africa

**Keywords:** physical abuse, sexual abuse, emotional abuse, high school violence

## Abstract

**Background:**

Gender-based violence (GBV) is a global public health issue, particularly affecting women and vulnerable youth, with an estimated 150 million girls and 73 million boys impacted worldwide. GBV can negatively impact the academic attainment of learners.

**Aim:**

To assess the experiences of GBV among high school-going youth in Estcourt, KwaZulu-Natal.

**Setting:**

This study took place in Estcourt, KwaZulu- Natal.

**Methods:**

An analytic cross-sectional study was conducted in four high schools among 349 learners who completed a standardised questionnaire. Data were analysed using the Statistical Package for the Social Sciences version 17. Descriptive analysis included a Chi-square test. A *p*-value ≤ 0.05 was considered statistically significant.

**Results:**

The study included 349 participants, comprising 195 girls (55.87%) and 154 boys (44.13%). More than half (53.01%) of the participants reported experiencing physical, emotional or sexual violence in their lives, including bullying, rape and discrimination. Chi-square tests were conducted to examine the associations between socioeconomic factors and experiences of GBV. The results showed a significant association between GBV and household education level (*p* < 0.005), household employment status (*p* < 0.02) and household occupation (*p* < 0.001). These findings suggest that socioeconomic conditions are significantly related to the prevalence of GBV among high school learners.

**Conclusion:**

This study highlights the prevalence of various forms of violence experienced by high school learners, emphasising the need for targeted interventions.

**Contribution:**

This study will provide relevant information that the Department of Education can use to develop and strengthen interventions for GBV in the school setting.

## Introduction

Gender-based violence (GBV) remains a critical public health and social issue worldwide, with particularly severe implications for high school-going youth in South Africa (Racionero-Plaza et al. 2021). The prevalence of GBV within educational environments not only affects the immediate safety and well-being of students but also has long-lasting impacts on their academic outcomes, mental health and overall development. Understanding the dynamics of GBV in this demographic is essential for informing effective interventions and policies aimed at reducing violence and promoting gender equality (Villardón-Gallego et al. 2023). South Africa has one of the highest rates of GBV in the world, rooted in a complex interplay of socio-cultural, economic and historical factors (Villardón-Gallego et al. 2023). High levels of unemployment, poverty and inequality exacerbate the vulnerabilities of young people, particularly those from marginalised communities (Bhana et al. 2021). Economic hardship can increase the vulnerability of young people to violence, as they may be forced into exploitative situations, including transactional sex or early marriage, as a means of survival (Muluneh et al. 2020). Moreover, limited access to education and health services restricts young people’s ability to seek help or escape abusive situations (Hulley et al. 2023). In many cases, the fear of stigma, social ostracism or retribution prevents victims from reporting incidents of violence, leading to underreporting and a lack of comprehensive data on the true scale of the problem (Hulley et al. 2023).

In addition, entrenched patriarchal norms and attitudes towards gender roles contribute to the normalisation of violence against women and gender minorities (Perrin et al. 2019). These norms often dictate rigid roles for men and women, leading to power imbalances that foster environments where violence against women and girls is normalised and, in some cases, even condoned (Mshweshwe 2020). Cultural practices such as child marriage, female genital mutilation and bride abduction further exacerbate the risk of violence for young girls in the region (Mshweshwe 2020). Furthermore, boys and young men are also not immune to GBV, with many experiencing physical and emotional abuse, often as a means of enforcing traditional notions of masculinity (Perrin et al. 2019). This culture of violence is often reflected in the high levels of intimate partner violence (IPV), sexual assault and femicide reported in the country (Maguele, Taylor & Khuzwayo 2023). Young people’s exposure to violence in South Africa begins at an early age, within the family or community, and continues throughout their adolescence and early adulthood.

Statistical data indicate that a significant number of South African youth experience various forms of GBV, including physical, sexual and emotional abuse (Govender 2023). For instance, national surveys reveal that a considerable percentage of adolescent girls report having faced sexual harassment or violence, both in and out of school settings. Boys, too, are not immune to violence, often experiencing bullying or coercive behaviours that can lead to harmful outcomes. In South Africa, individuals under 18 face high levels of violence. For instance, data from the ‘Birth to Twenty’ cohort study revealed that 48.0% of children had been exposed to violence in their communities, while 49.2% had experienced violence at home (Richter et al. 2018). Additionally, Meinck et al. (2016) found that 32.0% of children reported recurring abuse every month. The 2016 Optimus Study further revealed that 42.2% of nearly 4000 South African children had experienced some form of abuse, including sexual, physical, emotional and neglect-related abuse (Artz et al. 2018). School environments are not exempt from this issue. In a 2012 National School Violence Study examining over 6000 students from 121 South African schools, Burton and Leoschut found that 22.2% of students aged 12–18 years had experienced violence at school during the previous academic year (Milligan, Doss & Zungu 2024). Similarly, a study conducted in South Africa’s rural Eastern Cape province reported that 26.5% of Grade 10 girls had experienced multiple episodes of physical or sexual IPV, while 5.6% had been raped by a non-partner (Jewkes et al. 2019). Among boys in Grades 9–10, 31.8% admitted to committing physical or sexual IPV and 16.3% reported having raped a non-partner or participated in gang rape (Jewkes et al. 2019). Further research among South African adolescents aged 10–17 years estimated the lifetime prevalence of physical abuse at 56.3%, emotional abuse at 35.5% and sexual abuse at 9.0% (Jewkes et al. 2019). These experiences significantly shape young people’s perceptions of gender roles and relationships, potentially reinforcing cycles of violence (Murphy et al. 2021). The interplay of these experiences shapes their perceptions of gender roles and relationships, potentially perpetuating cycles of violence (Murphy et al. 2021).

The implications of GBV extend beyond immediate physical harm; they can affect academic performance, mental health and social development (Samakao & Manda 2023). Victims of GBV often report feelings of fear, anxiety and depression, which can hinder their ability to engage in educational activities. Research shows that exposure to violence during adolescence is linked to poor academic performance, increased school dropout rates and long-term psychological issues such as post-traumatic stress disorder (PTSD) (Hendricks 2019).

South Africa’s response to GBV has included implementing various policies and legislative frameworks to protect victims and prosecute perpetrators. However, challenges such as inadequate enforcement of laws, insufficient resources for support services and deeply ingrained cultural attitudes towards gender roles have limited the effectiveness of these measures (Calvino & Matadi 2023). Moreover, the coronavirus diseases 2019 (COVID-19) pandemic has exacerbated the situation, with lockdowns and economic disruptions leading to an increase in reported cases of GBV, particularly among young people who are often confined to abusive environments with limited avenues for seeking help (Mittal & Singh 2020).

Addressing GBV among youth in the sub-Saharan African region, specifically in South Africa, requires a multifaceted approach that includes strengthening legal frameworks, increasing access to education and economic opportunities, and challenging harmful cultural norms (Muluneh et al. 2020). It is also crucial to involve young people in designing and implementing interventions to prevent and respond to GBV, as their perspectives and experiences are essential to developing effective and sustainable solutions (Racionero-Plaza et al. 2021). By addressing the root causes of GBV and promoting gender equality, it is possible to create a safer and more supportive environment for all young people in the region.

### Aim

This study aims to assess the experiences of GBV among high school-going youth in Estcourt, KwaZulu-Natal province.

## Research methods and design

### Study design

A quantitative, cross-sectional study design was employed to assess the experiences of GBV among high school-going youth in Estcourt, KwaZulu-Natal province.

### Study setting

The study was conducted in four public high schools in Estcourt, KwaZulu-Natal, South Africa, within the Inkosi Langalibalele Local Municipality. A statistician was consulted and because of financial issues, a sample of four schools was recommended by the statistician, as long as the geographical representation of the schools in Estcourt was taken into account. The Inkosi Langalibalele Local Municipality is one of three that make up the uThukela District Municipality. This Municipality has 27 public high schools and 67 primary schools (Inkosi Langalibalele Local Municipality 2021). It also serves a population (230 924) that lives in rural, township and urban areas, and comes from a variety of ethnic backgrounds. Inkosi Langalibalele Municipality’s population is predominantly black African, accounting for 94.7%. Minority racial groups account for 5.3% of the total population (Inkosi Langalibalele Local Municipality 2021). More than half of the population is female (53.4%), with males accounting for 46.6%. Individuals under the age of 15 years account for 36.4% of the population, those between the ages of 15 and 64 years account for 59.6%, and those over the age of 65 years account for 4.0% of the population. IsiZulu is the most spoken language (93%.0) in this municipality (Inkosi Langalibalele Local Municipality 2021).

### Study population

The study population consisted of high school learners aged 16 years–20 years, from Grades 10 to 12, from four different high schools in Estcourt, KwaZulu-Natal. The participants were black Africans only. The study included both male and female participants.

### Sampling method and sample size

This study employed a stratified sampling technique. Out of the 27 public high schools in Estcourt, four were randomly selected from a list provided by the KwaZulu-Natal Department of Education. Proportionate sampling allocated learners to their respective schools based on their total enrolment. Participants were then stratified by sex to ensure a balanced representation of both sexes.

The sample size for this population-based study was determined by three main factors: the estimated prevalence of the outcome, a 95% confidence level and a 5% margin of error. On account of unknown prevalence rates for the primary outcomes, the researchers assumed a 50% prevalence of GBV among high school youth in Estcourt based on similar studies. This assumption led to an estimated sample size of 400 learners, but only 349 participated – 195 girls and 154 boys.

### Data collection

A researcher began by visiting the principals of the schools to ask for permission to collect data from their respective schools and discuss how the data would be collected. The researcher then met with the learners from each school to brief them on the study and answer their concerns. Recruitment of the participants began with an informational meeting held at each school after obtaining permission from the principal. The researcher was provided with a list of names from each grade to select the sample of learners. Meetings were held after school where eligible participants were informed about the study, their role in the study and invited questions. Those who were selected from the list of names were given a letter of information, a consent form, an assent form, a confidentiality statement and parental consent to sign. Data collection began on 01 February 2023 and continued until 05 March 2023. Written informed consent was obtained from parents or legal guardians of participants aged 16 years–17 years, as well as from participants aged 18 years and above. Assent was obtained from the respondents aged 16 years–17 years.

Data were gathered through a structured, standardised and self-administered questionnaire. The mode of data collection tool was a paper questionnaire, which was handed to the participants. The questionnaires were developed in the English language. The tool consisted of socio-demographics (section one), questions on experience of violence (section two) and on the contextual factors influencing GBV among high school-going learners (section three).

On the day of data collection, learners from each school were grouped in groups of 15 according to their grades in a large class where they answered the questions individually. Answering questions took roughly 30 min – 45 min. The researcher assisted learners who were having difficulty understanding the questions. Because of external issues such as not returning consent forms and parents refusing to allow their children to participate, only 349 learners comprising 195 girls and 154 boys were able to participate in the study.

### Statistical analysis

A quantitative, analytic, cross-sectional study was conducted in four high schools in Estcourt, KwaZulu-Natal, to assess the experiences of GBV among high school-going youth in Estcourt, KwaZulu-Natal province. The study included 349 students aged 16 years and older from Grades 10 to 12. A stratified random sampling technique was employed, and the required initial sample size of 400 participants was determined using the single population proportion formula, with a 95% confidence level and a 5% margin of error. Data were collected using a structured, standardised and self-administered questionnaire. Cronbach’s alpha was not utilised to determine the internal consistency of the measurement scale. Data analysis was conducted using the Statistical Package for the Social Sciences (SPSS) version 17 (SPSS Inc., Chicago, IL, US), with descriptive statistics and Chi-square tests performed. A *p*-value ≤ 0.05 was considered statistically significant.

### Validity and reliability

The validity and reliability of quantitative results are critical to the credibility of the research study (Sürücü & Maslakci 2020). The questionnaire was developed based on a comprehensive literature review and input from experts in intersectionality and violence studies, ensuring that the items accurately represent the constructs being measured. Further, a pilot test of the questionnaire with a small sample similar to the target population was conducted. This helped to identify ambiguous or confusing items and allowed for refinement. Cronbach’s alpha was not used to determine the reliability of the study.

### Ethical considerations

Ethical approval for the study was obtained from the University of KwaZulu-Natal’s Biomedical Research Ethics Committee (BREC) on November 21, 2022 (reference no: BREC/00003928/2022). Approval was also granted by the KwaZulu-Natal Department of Education (reference no: 2/4/8/4058). Permission to conduct research at specific school sites was obtained from the appropriate authorities. Participants aged 16 years-17 years provided written informed consent through their parents or legal guardians, whereas those aged 18+ gave consent directly. Participants’ rights and privacy were protected, with no names given in publications and simply codes used to identify them, maintaining secrecy. Pseudonyms were employed to further protect anonymity. All personal information recorded on the participant information sheet was kept strictly confidential.

## Results

### Socio-demographics of participants

Overall, 349 participants were enrolled in the study, with 195 females representing 55.87% and 154 males representing 44.13% of the population. The age of participants ranged between 16 years and 20 years. About half (52.15%) of participants in this study were between the age of 16 years – 17 years, 35.24% were in Grade 10. Over half of the participants, 235 (67.34%), were Zulu-speaking, and 128 (36.68%) of the participants were living with both parents, while 28.08% were living with their mothers only. Table 1 shows the socio-demographic characteristics of the respondents.

**TABLE 1:** Participant’s socio-demographics.

Items	Variables	*n*	%
Gender	Females	195	55.87
	Males	154	44.13
Age (years)	16–17	183	52.15
	18–20	166	47.56
Grade	Grade 10	123	35.24
	Grade 11	110	31.52
	Grade 12	116	33.24
Home language	Zulu	235	67.34
	English	102	29.23
	Xhosa	8	2.29
	Sestwana	2	0.57
	Afrikaans	2	0.57
Living with at-home	Both parents	128	36.68
	Fathers only	25	7.16
	Mothers only	98	28.08
	Foster parent	2	0.57
	Grandparents	43	12.32
	Extended Family	53	15.19
Housing	House	329	94.54
	Flat	19	5.46
Educational level (head of household)	**Primary school:**		
	Completed	296	84.81
	Incomplete	53	15.19
	**High school:**		
	Completed	208	59.60
	Incomplete	141	40.40
	**Diploma:**		
	Completed	68	19.49
	Incomplete	281	80.51
	**Degree:**		
	Completed	58	16.62
	Incomplete	291	83.38
	**Post Graduate:**		
	Completed	26	7.45
	Incomplete	323	92.55
Employment status (head of household)	Employed	180	51.58
	Unemployed	114	32.66
	Self-employed	55	15.76
Employment classification (head of household)	Professional	92	26.36
	Skilled	82	23.50
	Unskilled	175	50.14

*n*, number; %, percentage.

### Types of violence experienced by the participants

The participants reported having experienced various types of violence, including physical, emotional and sexual, and these experiences are presented and discussed in this section.

#### Experience of physical violence

About 164 participants in this study reported some form of physical violence, with 87 (53.05%) of females and 77 (46.95%) of males. However, female participants were more likely to report experience of physical violence than males. A greater proportion of males (84.4%) reported being slapped or hit with an object compared to females (74.7%). However, this difference was not statistically significant (*p* = 0.126). The percentages of males (79.2%) and females (77.0%) who reported being beaten up were similar, and the difference was not statistically significant (*p* = 0.73). A higher percentage of males (61.0%) indicated that they had been thrown something that could hurt, while 54.0% of females reported it. Nonetheless, this difference was not statistically significant (*p* = 0.365). A larger percentage of males (32.5%) reported experiences of being choked or attempting to drown compared to females (23.0%). However, this difference was not statistically significant (*p* = 0.175). The proportions of males (61.0%) and females (62.1%) who reported being punched, kicked or bitten were similar, and this difference was not statistically significant (*p* = 0.89). Both males (49.4%) and females (49.4%) reported being pushed or shoved at similar rates, and this difference was not statistically significant (*p* = 0.93). A higher percentage of males (49.4%) reported being hurt with a knife, gun or other dangerous weapons compared to females (37.9%), although this difference was not statistically significant (*p* = 0.177). Table 2 depicts the types of experiences of physical violence reported by the participants.

**TABLE 2:** Participants’ response to experience of physical violence (*N* = 164).

Event	Males (*n* = 77)		Females (*n* = 87)		Total	*p*-value
	*n*	%	*n*	%		
**Slapped, hit you with the object**						
Yes	65	84.4	65	74.7	130	0.126
No	12	15.6	22	25.3	34	-
**Beat you up**						
Yes	61	79.2	67	77.0	128	0.73
No	16	20.8	20	23.0	36	-
**Throw something that could hurt you**						
Yes	47	61.0	47	54.0	94	0.365
No	30	39.0	40	46.0	70	-
**Choke or attempt to drown you**						
Yes	25	32.5	20	23.0	45	0.175
No	52	67.5	67	77.0	119	-
**Punched, kicked or bite you**						
Yes	47	61.0	54	62.1	101	0.89
No	30	39.0	33	37.9	63	-
**Pushed or shoved you**						
Yes	38	49.4	43	49.4	81	0.93
No	37	48.1	43	49.4	80	-
**Used knife, gun, or another weapon**						
Yes	38	49.4	33	37.9	71	0.177
No	39	50.6	52	59.8	91	-

*N*, number; %, percentage.

#### Experiences of emotional violence

As depicted in Table 3, nearly equal percentages of males (55.8%) and females (57.5%) reported being threatened with harm, with no significant statistical difference (*p* = 0.7). A higher percentage of females (40.2%) received unsolicited letters compared to males (29.9%), although this difference was not statistically significant (*p* = 0.166). Similarly, more females (41.4%) reported receiving unsolicited phone calls than males (29.9%), but the difference was also not significant (*p* = 0.125). The percentages of males (29.9%) and females (26.4%) reporting unwanted items left for them were comparable, with no significant difference (*p* = 0.687). A larger percentage of males (45.5%) reported being followed compared to females (41.4%); still, this difference was not significant (*p* = 0.599). More females (72.4%) reported humiliation in front of others compared to males (66.2%), yet the difference was not statistically significant (*p* = 0.39).

**TABLE 3:** Participants’ response to the experience of emotional abuse (*N* = 164).

Event	Males (*n* = 77)		Females (*n* = 87)		Total	*p*-value
	*n*	%	*n*	%		
**Threatened to hurt you**						
Yes	43	55.8	50	57.5%	93	0.7
No	34	44.2	35	40.2	69	-
**Sent you unsolicited letters**						
Yes	23	29.9	35	40.2	58	0.166
No	54	70.1	52	59.8	106	-
**Made unsolicited phone calls to you**						
Yes	23	29.9	36	41.4	59	0.125
No	54	70.1	51	58.6	105	-
**Left you unwanted items**						
Yes	23	29.9	23	26.4	46	0.687
No	53	68.8	61	70.1	114	-
**Followed by someone with a car or on their feet**						
Yes	35	45.5	36	41.4	71	0.599
No	42	54.5	51	58.6	93	-
**Humiliated you in the presence of others**						
Yes	51	66.2	63	72.4	114	0.39
No	26	33.8	24	27.6	50	-
**Insulted or shame you**						
Yes	51	66.2	60	69.0	111	0.709
No	26	33.8	27	31.0	53	-
**Discriminated at school or home or community**						
Yes	45	58.4	55	63.2	100	0.53
No	32	41.6	32	36.8	64	-
**Share false accusations on social media**						
Yes	36	46.8	44	50.6	80	0.62
No	41	53.2	43	49.4	84	-
**Shared your naked photos on social media**						
Yes	14	18.2	10	11.5	24	0.23
No	63	81.8	77	88.5	140	-
**Been isolated from family**						
Yes	18	23.4	32	36.8	50	0.063
No	59	76.6	55	63.2	114	-
**Forced into an unwanted marriage**						
Yes	8	10.4	17	19.5	25	0.104
No	69	89.6	70	80.5	139	-

*N*, number; %, percentage.

A similar trend was observed in the reporting of insults, with 69.0% of females and 66.2% of males experiencing this, but the difference was insignificant (*p* = 0.709). More females (63.2%) reported discrimination at school, at home or in the community compared to males (58.4%), although this difference was not statistically significant (*p* = 0.53). A higher percentage of females (50.6%) reported being falsely accused on social media than males (46.8%), but again, this difference was not significant (*p* = 0.62). More males (18.2%) than females (11.5%) reported having their naked photos shared on social media, but this difference was not significant (*p* = 0.23). A greater proportion of females (36.8%) reported feeling isolated from their families, although this difference was not statistically significant (*p* = 0.063). Finally, more females (19.5%) reported being forced into an unwanted marriage compared to males (10.4%), with no significant gender difference noted (*p* = 0.104).

Overall, while certain trends suggest higher reports of specific experiences among females, most differences between genders were not statistically significant, indicating that both males and females encounter various forms of violence and harassment.

#### Experiences of sexual abuse

Table 4 reveals the patterns of sexual violence, with similar experiences reported by both males and females across various forms of sexual coercion and violence. Approximately 28.6% of males and 29.9% of females reported being forced to have sex, with a *p*-value of 0.779 indicating no significant gender difference. Both genders reported similar experiences, with 35.1% of males and 35.6% of females having someone expose their sexual organs; the *p*-value of 0.94 shows no significant difference. A higher percentage of females (46.0%) reported being forced into unwanted acts such as kissing compared to males (32.5%). Although the *p*-value of 0.078 suggests a trend towards significance, it is not statistically significant. The rates of unwanted kissing were also similar, with 62.3% of males and 67.8% of females reporting such experiences and a *p*-value of 0.46, indicating no significant difference. About 50.6% of males and 52.9% of females reported unwanted touching of private parts, with a *p*-value of 0.776 showing no significant difference.

**TABLE 4:** Participants’ response to the experience of sexual abuse (*N* = 164).

Event	Males (*n* = 77)		Females (*n* = 87)		Total	*p*-value
	*n*	%	*n*	%		
**Been forced to have sex**						
Yes	22	28.6	26	29.9	48	0.779
No	55	71.4	59	67.8	114	
**Show off his or her sex organs to you**						
Yes	27	35.1	31	35.6	58	0.94
No	50	64.9	56	64.4	106	
**Been forced into unwanted sex acts, kissing**						
Yes	25	32.5	40	46.0	65	0.078
No	52	67.5	47	54.0	99	
**Tried to kiss you without your permission**						
Yes	48	62.3	59	67.8	107	0.46
No	29	37.7	28	32.2	57	
**Try to touch your breast, buttocks, vagina or penis**						
Yes	39	50.6	46	52.9	85	0.776
No	38	49.4	41	47.1	79	
**Put fingers or objects in your vagina or penis**						
Yes	21	27.3	22	25.3	43	0.773
No	56	72.7	65	74.7	121	
**Been forced by your girlfriend to impregnate her**						
Yes	9	11.7	9	10.3	18	0.784
No	68	88.3	78	89.7	146	
**Ever been forced by your boyfriend to get pregnant?**						
Yes	5	6.5	6	6.9	11	0.918
No	72	93.5	81	93.1	153	
**Force someone or being forced to have an abortion**						
Yes	5	6.5	3	3.4	8	0.366
No	72	93.5	84	96.6	156	
**Force someone or being forced to stop using birth control**						
Yes	6	7.8	4	4.6	10	0.394
No	71	92.2	83	95.4	154	
**Been restricted from using a condom**						
Yes	9	11.7	5	5.7	14	0.174
No	68	88.3	82	94.3	150	

*N*, number; %, percentage.

The percentage of males (27.3%) and females (25.3%) experiencing unwanted insertion was comparable, with a *p*-value of 0.773 indicating no significant difference. Reports of being forced to get pregnant or for impregnating someone were similar, with 11.7% of males and 10.3% of females affected and a *p*-value of 0.784 indicating no significant difference. About 6.5% of males reported forcing their girlfriends to abort and 3.4% of females reported being forced to have an abortion, the *p*-value of 0.918 indicates no significant difference. The percentage of being forced to stop using birth control was 7.8% for males and 4.6% for females, with a *p*-value of 0.394 indicating no significant difference. Finally, 11.7% of males and 5.7% of females reported restrictions on condom use, with a *p*-value of 0.174 suggesting no significant difference.

Overall, while some trends indicate higher experiences among females, most differences in reported experiences of sexual coercion and violence between genders were not statistically significant, highlighting a shared prevalence of these issues across both groups.

### Comparison of the demographic factors influencing gender-based violence among high school-going youth

Both male and female participants reported experiencing GBV; however, there was no significant association with age (*p* = 0.269), gender (*p* = 0.32) or type of household dwelling (*p* = 0.9). Among participants, only 24.5% from households with less than primary education reported experiencing violence. In comparison, 48.9% of those from primary-educated households, 50.5% from secondary-educated households and 53.3% from post-secondary-educated households reported similar experiences. The analysis yielded a *p*-value of less than 0.005, indicating a significant association between household education level and experiences of violence. This suggests that higher educational attainment correlates with an increased likelihood of reporting such experiences. Table 5 shows the relationship between the socio-demographic distribution of the participants and violence act.

**TABLE 5:** Socio-demographic factors associated with sexual, physical and emotional violence (*N* = 349).

Items	Yes (*n* = 185)		No (*n* = 164)		Total	*p*-value	Interpretation
	*n*	%	*n*	%			
**Sex**							
Male	77	50.0	77	50.0	154	0.32	-
Female	87	44.6	108	55.4	195	-	-
**Age group (years)**							
16–17	80	44.0	102	56.0	182	0.269	Not one student missing the age
18–20	83	50.0	83	50.0	166	-	-
**With whom do you live**							
Both parents	53	41.4	75	58.6	128	0.2	-
Father	16	64.0	9	36.0	25	-	-
Mother	42	42.9	56	57.1	98	-	-
Grandparents	24	55.8	19	44.2	43	-	-
Extended family	28	52.8	25	47.2	53	-	-
Other	1	50.0	1	50.0	2	-	-
**Dwelling**							
House	155	47.1	174	52.9	329	0.9	-
Flat	9	47.4	10	52.6	19	-	-
**Education of HH**							
< primary	13	24.5	40	75.5	53*	< 0.005	There is an association between education of HH and violence
Primary	43	48.9	45	51.1	88	-	-
Secondary	52	50.5	51	49.5	103	-	-
post-secondary	56	53.3	49	46.7	105	-	-
**Employment HH**							
Employed	91	50.6	89	49.4	180*	< 0.001	There is a significant association level of employment of HH and violence
Unemployed	39	34.2	75	65.8	114	-	-
Self-employed	34	61.8	21	38.2	55	-	-
**Occupation HH**							
Professional	53	57.6	39	42.4	92*	< 0.02	There is a significant association between the occupation of HH and violence
Skilled	41	50.0	41	50.0	82	-	-
Unskilled	70	40.0	105	60.0	175	-	-

* , Statistically significant.

Level of significance *p* < 0.05.

HH, head of household; *N*, number; %, percentage.

## Discussion

The study aimed to assess the experiences of GBV among high school-going youth in Estcourt, KwaZulu-Natal province. In this study, the experience of GBV was 53.01%. Globally, exposure and experience of GBV among high school-going youth have been under intense review. Although GBV has been reported for more than decades, young people continue to be either victims or perpetrators of violence (Mahlori, Byrne & Mabude 2018).

This study found that both male and female participants experienced various forms of violence, including physical, emotional and sexual abuse. A significant majority of young males (79.2%) and females (77.0%) reported being beaten by someone, while 84.4% of males and 74.7% of females indicated that they had been intentionally struck with an object. Supporting these findings, Jewkes et al. (2019) reported that approximately 22 of learners experienced physical assault at school, highlighting the pervasive nature of violence in South African educational settings. This violence is often exacerbated by broader societal issues such as poverty, inequality and community violence. Furthermore, the 2016 South African School Violence Study emphasised that violence in schools is not only widespread but also frequently underreported because of fears of retaliation or a lack of trust in authorities (Burton 2016).The prevalence of physical violence is associated with numerous negative outcomes for adolescents, including mental health issues, academic underachievement and an increased risk of engaging in violent behaviour (Malongo & Mwale 2019).

In addition, this study revealed that both boys and girls reported significant experiences of emotional abuse, with a higher percentage of girls (72.4%) than boys (66.2%) indicating feelings of humiliation in the presence of others. This disparity suggests that girls may experience emotional abuse more intensely or frequently than boys, pointing to potential gendered patterns in the expression and perception of emotional abuse (Chaplin 2015). The data also showed that a larger proportion of girls (63.2%) reported experiencing discrimination at school, at home or within their community compared to boys (58.4%). This indicates that girls may face more frequent or severe discrimination in the school environment (Chaplin 2015).

Furthermore, students reported experiencing bullying, which remains a significant issue in South African schools, with alarmingly high prevalence rates (SACAP 2016). Studies indicate that a large percentage of students endure various forms of bullying, including physical, emotional and cyberbullying (SACAP 2016). Notably, passive forms of bullying, such as exclusion and spreading rumours, are more common among girls, whereas boys tend to engage more frequently in active bullying. Research highlights that ongoing exposure to violence and discrimination in various settings, including schools, contributes to this pervasive problem (SACAP 2016). The high rates of bullying correlate with broader societal violence, underscoring the urgent need for effective interventions and policies aimed at creating safer school environments.

This study reveals alarmingly high levels of sexual abuse among both young male and female students, with approximately 62.3% of young men and 67.8% of young women reporting being kissed without their consent. In addition, 50.6% of boys reported instances of non-consensual touching of their buttocks or penis, while 52.9% of girls indicated unwanted touching of their breasts, buttocks or vagina. Despite these disturbing figures, the research did not find a significant association between gender and the likelihood of experiencing such violent acts. This suggests that both male and female students are equally vulnerable to sexual abuse, challenging common perceptions that often frame girls as the primary victims.

The pervasive nature of sexual abuse in high school environments is deeply troubling and carries far-reaching consequences for individuals and society as a whole. Research consistently indicates that sexual abuse is alarmingly prevalent in educational settings, reflecting broader societal issues such as gender inequality, power dynamics and the lack of effective protective measures for young people (Burton 2016). In South Africa, for instance, a study conducted by the Centre for Justice and Crime Prevention (CJCP) found that 36.3% of high school learners reported experiencing some form of sexual abuse in their lifetime. These findings align with global trends, where school environments often become hotspots for sexual harassment and abuse, exposing learners – particularly girls – to threats from peers, teachers and other school staff (Leoschut & Makota 2016). The normalisation of such behaviours, coupled with inadequate reporting mechanisms and support systems, exacerbates the issue, leaving many victims feeling isolated and unsupported.

This study highlights the urgent need for comprehensive strategies to address and combat sexual abuse in schools (Schneider & Hirsch 2020). Educational institutions must prioritise the development of robust policies that promote a safe and respectful environment for all students. Effective training for staff and students on consent, healthy relationships and reporting procedures is essential. Furthermore, creating accessible support systems can empower victims to come forward, fostering a culture of accountability and respect. Addressing these challenges is crucial for the well-being of individual students and the health of the broader educational community.

The lack of significant association between gender (*p* = 0.32) and age (*p* = 0.269) with experiences of GBV is notable. This finding suggests that the prevalence of GBV is not differentiated by these demographic variables within the study population. Previous research has shown that gender norms and societal expectations can influence experiences of violence (Laurenzi et al. 2024); however, this study indicates that high school youth may experience GBV regardless of these factors. This could imply that the social environment surrounding these youths may contribute equally to their vulnerability to violence, irrespective of their age or gender.

The absence of a significant relationship with age further suggests that GBV is a pervasive issue that transcends developmental stages in adolescence (Laurenzi et al. 2024). This is particularly concerning, as it highlights the necessity for comprehensive educational programmes on GBV that address all youth, irrespective of age. Future research should further explore the contextual factors that might contribute to these experiences, potentially examining the role of peer dynamics and school environments.

A striking finding from this study is the significant association between household education level and experiences of GBV, with only 24.5% of participants from households with less than primary education reporting violence, compared to 53.3% from post-secondary educated households (*p* < 0.005). This suggests that educational attainment within households plays a critical role in shaping attitudes and awareness regarding GBV.

Higher educational levels may correlate with greater access to resources and knowledge about rights, leading to increased violence reporting (Vanner, Holloway & Almanssori 2022). Youths from better-educated households may be more likely to recognise and articulate experiences of GBV, while those from less educated backgrounds may either lack awareness or feel disempowered to report such incidents. This finding aligns with existing literature, which posits that education can empower individuals to challenge violent behaviours and seek help (Vanner et al. 2022).

The study also revealed a significant association between head of household employment status and experiences of GBV, with 50.6% of participants from employed households reporting violence, compared to 34.2% from unemployed households and, notably, 61.8% from self-employed households (*p* < 0.001). These findings suggest that the dynamics of household employment may influence the prevalence of GBV among youth. One possible explanation is that self-employment might be associated with unstable financial situations, leading to increased stressors within the household that could escalate tensions and result in violence (Judd et al. 2023). Conversely, stable employment might provide a framework for support and resources that could mitigate the risk of violence. However, it is important to consider that employed individuals may still encounter societal pressures and stressors that contribute to violence (Martins, Matos & Sani 2023).

Finally, the significant association between head of household occupation type and experiences of GBV (*p* < 0.02) indicates that individuals from professional households reported a higher prevalence of violence (57.6%) compared to those from skilled (50.0%) and unskilled (40.0%) households. This finding suggests that the nature of employment may intersect with other socio-cultural factors that influence the prevalence of GBV.

Professionals may experience unique stressors related to their work environments or may be more aware of GBV resources and, thus, more likely to report experiences of violence. Conversely, unskilled labourers may face different societal challenges that impact their reporting behaviour. This highlights the need for targeted interventions that consider occupational dynamics and their impact on youth experiences of GBV. This study underscores the importance of socio-economic factors in understanding GBV among high school students. While age and gender did not significantly influence experiences of violence, household education level, employment status and occupation type emerged as crucial determinants. These findings call for comprehensive, intersectional approaches to prevent GBV, emphasising the need for educational initiatives that empower all youth, regardless of their socio-economic background. Future research should continue to explore the complex interactions between these factors and the broader societal context in which youth live, ultimately informing policies aimed at reducing the prevalence of GBV.

### Limitations of the study

The findings are limited because of the sample being restricted to youth in Grades 10–12, aged 16 years – 20 years. This restriction may introduce selection bias, as the study did not account for learners absent from school during the research period. Consequently, the selection of participants was confined to those present during recruitment, potentially overlooking individuals whose absence could be related to the issues under investigation.

With only four schools participating in the study, the generalisability of the findings to all high school students in Estcourt or KwaZulu-Natal is limited. In addition, the study’s cross-sectional nature precludes any assessment of trends or changes in the incidence of GBV among high school students over time. Despite these limitations, the study’s findings serve as a foundation for generating hypotheses for future research.

### Recommendations

Based on the findings, more than half of this study’s participants have encountered violence (physical, sexual or emotional), which can have serious physical and mental health consequences including poor academic performance. Furthermore, the findings underline the importance of comprehensive implementation of evidence-based preventive measures and institutional initiatives to minimise violence against young boys and girls. To address negative cultural norms that contribute to violence, initiatives must involve stakeholders from all levels of the ecological framework, including very young adults. These programs should emphasise learners’ roles as peer advocates against violence, raise awareness, and develop shared standards that encourage nonviolent behaviour.

## Conclusion

Gender-based violence is prevalent among high school learners in KwaZulu-Natal, with both boys and girls experiencing various forms of abuse early in life, leading to potential physical, mental and academic challenges. While the findings may not be generalisable to all South African high school learners, they underscore the significant issue of violence among young people in the region. Socio-economic factors, including household education, occupation and employment levels, play a key role in influencing aggressive behaviour. Addressing GBV in schools requires a comprehensive, whole-school approach that involves management, learners and the curriculum to ensure consistent and reinforced messaging.
